# Factors influencing acceptance or decline of a hospital-sponsored scholarship by nursing students in Taiwan: a qualitative descriptive study

**DOI:** 10.1186/s12912-021-00547-w

**Published:** 2021-02-06

**Authors:** Chang-Ting Tsou, Kuan-Ling Chen, Yun-Fang Tsai

**Affiliations:** 1 Graduate Institute of Clinical Medical Sciences, College of Medicine, Chang Gung University, 259, Wen-Hwa 1st Road, Tao-Yuan, 333 Taiwan (ROC); 2 School of Nursing, College of Medicine, Chang Gung University, 259, Wen-Hwa 1st Road, Tao-Yuan, 333 Taiwan (ROC); 3 Department of Nursing, Chang Gung University of Science and Technology, Tao-Yuan, Taiwan (ROC); 4 Department of Psychiatry, Chang Gung Memorial Hospital at Keelung, Keelung, Taiwan (ROC)

**Keywords:** Career planning, Hospital-sponsored scholarship, Nursing school graduates, Nurse retention

## Abstract

**Background:**

Maintaining sufficient nursing personnel is critical for healthcare systems worldwide. Improving retention of nurses is one means of addressing this shortfall. To foster retention, some hospitals in Taiwan provide nursing scholarships contingent on recipients signing a 3-year employment contract. However, it is unclear what factors influence students’ decisions to accept or reject a scholarship.

**Methods:**

The purpose of this exploratory qualitative descriptive study was to obtain an understanding of the subjective experiences of fourth-year nursing school students (*N* = 87) who accepted (*n* = 43) or declined (*n* = 44) a hospital-sponsored nursing school scholarship. Students were selected by purposive sampling from the department of nursing of a private university in northern Taiwan. Data were collected between 2013 and 2014 using face-to-face-in-depth interviews.

**Results:**

The mean age of participants was 22.7 years; most (94%) were female (*n* = 82). Analysis of the interview data showed the choice to accept or decline the scholarship and making career decisions occurred in three stages for both groups: the considering their options, making the decision, and assessment of their decision.

**Conclusions:**

Although the variables at each of these stages differed between groups, both sponsored and non-sponsored students felt a responsibility to continue as employees of the hospital after graduation. Financial status, the hospital environment, and future long-term career goals were important factors affecting the acceptance or rejection of the hospital scholarship. These results could provide insight into factors students consider important for making long-term commitments as a nursing professional, which could not only improve retention of nurses, but also serve as a guideline for career planning.

## Introduction

The lack of nursing manpower remains a critical issue of concern in the global healthcare system [1]. Many studies confirm that the shortage of nurses greatly impacts not only the quality of patient care but also the health of patients as a result of smaller hospital health management teams [2, 3]. Retention of nursing professionals is on the decline, making recruiting and retaining nurse specialists a chronic international challenge [4–8]. Countries in North America and Europe have reported resignation rates of nursing specialists ranging from 14% to as high as 49% [1, 4], and new nurse graduates have the lowest retention rate [9]. Therefore, any means of incentivizing retention of nurses could help improve the quality of patient care. One means of retaining nurses is helping them transition from nursing students to nursing professionals [9].

## Background

Nursing school is designed to prepare new graduates for employment in clinics and hospitals. However, when new graduates enter the workplace they are faced with transitioning to real-life situations that are stressful and challenging. Lack of self-confidence, the unfamiliar demands and procedures required in their new environment can make nurses more likely to consider a different career [10–14].

When new graduate students make career decisions, positive attitudes towards their decision have been have been shown to be positively correlated with retention [15]. Positive attitudes towards a nursing career are determined by students’ professional interests as well as confidence in their clinical skills [15, 16], which can be influenced by individual instructors, career organizations, and support from family and friends [17, 18]. In addition, individual characteristics, abilities, learning experiences and work skills also contribute to a person’s career decisions [18–20].

Nurses comprise the largest component of the healthcare system, and inadequate staffing affects hospital performance and quality of patient caring [19]. Several approaches for increasing retention of nurses are aimed at new nurse graduates, which include enhancing employee benefits, and incentivizing undergraduate students by offering paid employment [10, 13].

In Taiwan, the employment rate of nursing professionals is 58.2%, while the resignation rate during the first 6 months of employment is 60 and 27.8% within a year; this rate is two to three times higher than for nurses in Japan or Singapore [21–23]. A survey of nurses Taiwan found clinical nurses considered quitting (62%) or changing jobs (64%) [24]. Although the number of nursing graduates in Taiwan increases every year, the retention rate of new nurses remains low. Therefore, there is an urgent need to determine how to effectively retain newly recruited nurses in the clinical workplace.

Programs promoted to increase retention by Taiwan’s healthcare industry include government-funded rural-area plans and hospital-sponsored scholarships offered in collaboration with nursing schools. Collaborations between industry and academia can help smooth the transition of new nurses from student to professional nurse [14]. A program in Taiwan, titled the “The Last Mile”, was designed to bridge the gap between classroom skills and clinical practice, with the goal of reducing nurse turnover [14, 25]. However, whether this type of program encourages students to pursue careers as professional nurses has not been examined.

This study aimed to explore the experiences of nursing students who had decided to accept or decline participation in a hospital-sponsored scholarship program that would begin following graduation. Our findings could provide nurse educators with information about the components of transition programs that are important for encouraging nursing students to choose a career in clinical nursing.

## Methods

### Study design

This exploratory qualitative descriptive study was conducted using face-to-face semi-structured interviews, to obtain an understanding of the subjective experiences of fourth-year nursing school students who had decided to accept or decline participation in a hospital-sponsored scholarship program that would begin following graduation. Data were collected between 2013 and 2014.

### Participants

All procedures in this study were approved by the Institutional Review Board (IRB) in accordance with the ethical standards of the 1964 Helsinki declaration and its later amendments. Following ethics approval (IRB 102-2529B), nursing students scheduled to graduate were recruited from a private school of nursing in northern Taiwan, which is affiliated with a college of medicine. This is a 4-year baccalaureate program for students who have completed a high school education. Students are admitted through a high school entrance examination, admission application and recommendations. The school was chosen because it is where the sponsored scholarship was implemented. The scholarship program is open for applications in to senior students in November (8 months before graduation). Nursing students who met the qualifications for the sponsored scholarship were recruited by purposive sampling 2 months prior to graduation. Students were eligible to participate in the study if they met the following inclusion criteria: at least 20 years of age; had chosen to accept or decline the hospital-sponsored scholarship prior to graduation from the school of nursing in year 2013 or 2014; of clear mind; and able to communicate in both Standard Mandarin and Taiwanese Mandarin. Students suffering from psychological problems or emotional distress in the past year were excluded from the study.

### The hospital-sponsored scholarship

It is not uncommon for departments of nursing at universities and some technical and vocational nursing schools in Taiwan to provide scholarships for nursing students. However, the Private University School of Nursing (SN-A) made the decision to implement a hospital-sponsored scholarship for the first time in 2013, in collaboration with a private medical center (H-A). The university and the hospital were founded by the same corporation. This type of collaboration, also known as industry-academia cooperation, was proposed by the hospital and this was the first time the program was implemented at the nursing school. The total amount of the scholarship differs with the type of contract, which is either for three-years or five-years. Students are recruited 1 year before graduation, and the school and hospital offer an orientation program, which include hardcopy handouts as references. Applicants to the program must have an average grade of at least 75 (GPA 3.0) for academic courses and their conduct grade must be above 80.

### Data collection

Data were collected 2 months prior to graduation from nursing school between 2013 and 2014. Students who met the inclusion criteria and agreed to participate were contacted by telephone to determine a convenient time and place for the interview. Individual semi-structured face-to face interviews were conducted by the first and the second authors and audio recorded with permission from the participants. Interviews were conducted in a quiet place and lasted from 25 to 35 min; only one interview was conducted with each participant. Both interviewers had master’s degrees and had completed advanced courses in qualitative studies. Two different semi-structured interview guides were used, depending on whether the participant was a hospital sponsored or non-hospital sponsored nursing student (Table 1).

**Table 1:** Semi-structured interview guides for nursing students who decided to accept or decline the hospital-sponsored scholarship

Scholarship status	Questions
Scholarship accepted	1. What are your thoughts on the hospital scholarship system for nursing students?
	2. Why did you choose to participate in the program? Follow-up questions: What factors did you consider? Did you discuss this with anyone? What was their point of view?
	3. What is your expectation for the hospital sponsored scholarship?
	4. As a hospital sponsored student, what concerns you and your family the most? Follow-up questions: What did you do to figure it out and find the solution to it?
	5. Any thoughts or suggestions you would like to share based on your experience of being a hospital sponsored student?
Scholarship declined	1. What are your thoughts about the hospital sponsored scholarship system for nursing students?
	2. What are the reasons you chose not to participate in the program? Follow-up questions: Did you discuss this with anyone? If yes, what was their point of view? Did you attend any school-sponsored seminars introducing this program?
	3. If you had a second chance, would you choose to participate in the scholarship program? What would be the reason for your choice?
	4. Is there anything you would like to share or suggest regarding about being a non-sponsored student?

Demographic data were collected using a survey questionnaire. Interviews continued until data saturation was reached, which occurred when no new categories were presented. For the hospital-sponsored students, data saturation was reached with the sixteenth participant; for non-sponsored students, saturation was reached with the fourteenth participant. This was the first stage of a long-term study; the second stage of qualitative assessments are planned to be conducted at 5-years following graduation. In order to account for attrition over a 5-year period, a minimum of 40 students in each group were interviewed to ensure an adequate sample of participants will be available for the second stage of interviews.

### Data analysis

Audio-recorded interview data were transcribed verbatim as word files and analyzed using content analysis [26, 27]. Coding and analysis were performed with Atlas.ti computer software (Version 5.2). First, the software program organized the data into different codes. Next the codes were grouped by similar relationships to form categories. The different categories comprised an overall themes and subthemes describing the experiences of the students as they made their decision to accept or decline the hospital-sponsored scholarship.

Once data analysis was complete, relevant quotes were identified by the first author. The meaning of the quotes, rather than word-for-word translations, is more important in qualitative studies. Therefore, the relevant quotes were translated from Mandarin to English by a translator proficient in Mandarin and English. The translations were then back translated to Mandarin by the third author, in order to ensure the meaning of the translation from Chinese to English had been retained. The translations were finalized following discussions between the first and third author.

### Rigor

Trustworthiness of the data was guided by the four criteria of Lincoln and Guba [26], credibility, dependability, confirmability and transferability. Credibility was established by prolonged engagement with the nursing students prior to graduation. Member checking was conducted with participants who had accepted (*n* = 3) and declined (*n* = 3) the hospital-sponsored scholarship to further increase credibility of the data. The first author presented the preliminary interpretation of the verbatim transcribed interview text to confirm the accuracy of the findings. If a participant disagreed with the researchers’ findings, the participant provided their interpretation, and the researchers revised the findings accordingly. Credibility was maintained by the experience of all authors in conducting and analyzing the research and peer debriefing with two PhD candidates experienced in both qualitative and quantitative research and one PhD candidate with experience in quantitative research. Dependability was established by determining reliability of the data using the method of Miles and Huberman [27], which examines if the study findings are consistent among researchers. After the first peer debriefing, reliability was 85.2%; after the second debriefing a reliability of 95% was achieved. Dependability was further enhanced by maintaining an audit trail, which included audio recordings, rich descriptions of the study methods, and detailed text notes of data collection for data verification and comparison. The audit trail is archived for 5 years. Confirmability of the data was maintained by the use of reflexive journals and weekly meetings regarding the applicability of categories and themes, and through discussions about the findings among all authors, which continued until consensus was reached. Transferability was established by purposive sampling of participants, and data saturation.

## Results

### Participants

A total of 87 students were interviewed for this study; the mean age was 22.07 years (SD = 0.23) and 82 were female. A total of 43 students had elected to accept the hospital-sponsored scholarship (HS); 44 students declined, and we regarded them as non-hospital sponsored (nHS). The HS and nHS groups were equally distributed in terms of sponsorship and year of graduation (Table 2). Students who graduated in 2013 are identified as HS1 or nHS1; those who graduated in 2014 are identified as HS2 or nHS2.

**Table 2:** Characteristics of the nursing school students (*N* = 87) who accepted and declined the hospital-sponsored scholarship

	Hospital-sponsored scholarship	
	Accepted (*n* = 43)	Declined (*n* = 44)
Gender, n (%)		
Female	40 (93)	42 (95)
Male	3 (7)	2 (5)
Age, (years), mean (SD)	22.07 (0.26)	22.07 (0.33)
Year of graduation, n (%)		
2013	25 (58)	21 (48)
2014	18 (42)	23 (52)

*Abbreviation*: *SD* Standard deviation

### The decision-making process

Analysis of the interview data showed the decision-making process involved three stages for both HS and nHS. These stages of deciding to accept or reject the scholarship could be described with three themes: 1) considering options, 2) making the decision, and 3) analyzing their decision. Accepting or rejecting the offer of a scholarship was a major decision that would have an impact on new nursing students’ post-graduation commitments. The subthemes and subthemes of the three stages of the decision-making process are summarized in Table 3.

**Table 3:** Themes and subthemes describing the three stages of the decision-making process for accepting or declining the hospital-sponsored scholarship

Stage	Theme	Subtheme
1	Considering options	Emotional reactions
		Information gathering
2	Making the decision	Financial evaluation
		Professional evaluation
		Evaluation of the environment
		Evaluation of employment outcomes
3	Analyzing the decision	Anxiety and uncertainty
		Coping with the decision

#### Considering the options

Both groups of students reported that the program prevented them from assessing the benefits of the scholarship. All participants struggled to make an informed decision to sign or not to sign the contracts because this program was new to the nursing school. Therefore, initially they needed to consider their options and weigh the pros and cons of the program. The absence of feedback from other students made it difficult to assess how a scholarship that was tied to a job commitment would impact their education and careers as nurses. This preliminary stage of considering their options involved emotional reactions and information gathering.

##### Emotional reactions

Both HS and nHS students reacted with shock and doubt upon first hearing of the program. They thought this kind of proposal was too good to be true, and suspected there were undisclosed expectations. These reactions were reflected be three students in the sponsored group. One student (HS1-17) said, “I think this is great, but I still have doubts that this is a trap or something. Why all of a sudden are there quotas for hospital-sponsored students?” Another participant (HS2-30) said, “No way! How could there be such a wonderful thing in the world. This must be some sort of trick by the corporation. You never know what they are really up to.” Another said,

When I first heard this, I thought it was too good to be true. They offered you a great deal of money and a solid job. Turns out they just want you to sign a contract that you can never escape. (HS2-32)

A non-sponsored student said, “No, I don’t believe it (it sounds too easy). No Pain, no gain.” (n HS1-5).

##### Information gathering

Information gathering was described by 85% of the sponsored students, and 64% of the non-sponsored students, which involved trying to better understand how the hospital-sponsored scholarship was structured. Students attended information seminars and asked a lot of questions. They also had discussions with former teachers, close friends, and family to determine if the program would be to their benefit. In general, students in the HS group received positive opinions from others, as described by the following quotes:

I personally went back to my senior high school and asked the opinion of my high school teacher. She told me he had been a sponsored student [in college] and thought this was a great opportunity. In addition to that, my family all thought it was OK to do so; they felt it was good to not only get a guaranteed job but also an extra upfront payment (HS1-17).

I discussed [the program] with my boyfriend first because he was also in Taipei and he was a new graduate. I thought he would know better than I did. Then I talked to my family about this and they were all supportive. They thought there were dormitories in the hospital (about a 5-10-minute walk to the hospital) and I was very familiar with the environment. (HS1-5)

One student described the difficulty many students had in getting enough information to decide because the description of the program provided by the nursing school was vague and resorted to obtaining more details from the hospital.

Initially, I went to the seminars, but they did not explain the details (of the program) very well. I discussed this with other classmates but still had doubts. Finally, many of us went directly to the hospital. Some students asked the school to deliver questions to the hospital. Once I knew more, I felt more confident and relieved. (HS2-30)

When the program was offered for the second time in 2014, some students who did not feel the information sessions provided enough detail were able to talk to the 2013 graduates.

We were the second year of graduates [eligible] for this program. Therefore, I asked for the opinions of some seniors from the first year in advance. They all told me this was quite good and with the extra money they felt less pressure when it came to living costs or the student loans. I also thought this was a good decision to make. (HS2-26)

Non-sponsored students did not report seeking additional information about the program. Their information gathering was focused on feedback from family, as described one participant (nHS1-2):

I discussed this with my family, but in the end, it was me who needed to make the final call. My family let me make my own decision and my father thought it would be better to stay as close as possible to my home. This was because a nursing professional is not a fixed working-hour job and you have to work in different shifts. So, my parents hoped if I could stay close to home it would be more convenient for them to take care of me.

#### Making the decision

Students made the final decision to accept the scholarship by setting aside their emotional feelings and rationally evaluating the impact of the program on their lives. They considered how their decision would affect them financially and professionally, and evaluated the work environment and job expectations.

##### Financial evaluation

More hospital-sponsored students (76%) considered the financial impact as important for their decision compared with 54% of the non-sponsored students and was based on their current financial status. Students elected to participate in hospital-sponsored scholarship program in order to pay-off their student loans and reduce their financial burden. One student (HS1-4) said, “Well, I just wanted to sign the contract. I have student loans from four years of my university life. It will be great if I have money to pay back the loans as soon as I graduate.” They also made decisions by evaluating the financial benefits and drawbacks of signing the contract or breaking the agreement, which was described by another student who elected to take the scholarship:

I signed the five-year contract and, as a result, I will get an extra $370,000 [NTD]. If I break the agreement, there is a formula to calculate the amount you need to repay using the bank’s rate. After doing the math, I thought it was reasonable, so I signed it. (HS1-3)

Although non-sponsored students also evaluated the financial impact of their decision, they were more likely to consider the effect on their future and less likely to describe worries about income. They viewed the requirement of a contract as limiting future career choices. One student (nHS2-34) said, “I thought it was not a good deal. You need to pay back the money if you quit the job before the contract ends, which would add pressure (to stay).” Another student had a similar perspective:

My family’s financial status is not bad. I don’t want to be bound by the contract; getting the money first would keep me from getting out of it (the contract). There are still many things one can do in a lifetime and it is not worth being trapped. (nHS2-25)

##### Professional evaluation

Professional evaluation was part of the decision-making process for 51% of the hospital sponsored students compared to 69% of the non-sponsored students. The evaluation was primarily whether accepting the scholarship would provide placement in a setting that was viewed as desirable. Hospital-sponsored students viewed placement in a clinical environment and establishing a good clinical foundation as their top priority after graduation. Professionally, the scholarship program was a win-win situation: they would gain experience and money at the same time. One student (HS1-3) thought accepting the scholarship would be better than working and later moving to the United States for additional training, she said, “Originally, I planned to work for two or three years, and then go to America to get a RN license or something like that.” Another student said:

Actually, my father asked me to keep studying for a higher degree, but I told him that if I keep studying but without working in a clinical environment, I would not feel the spirit of nursing and everything would be just theoretical and not practical. I’ve convinced them that I should have some working experience in a clinical environment first. The scholarship was another reason to push me to work hard and learn more in clinical environment since I already wanted to work there and there will be extra money from the scholarship. (Student HS1-9)

Many of the non-sponsored students had already made plans for their professional future and believed signing the contract would be stressful and an emotional burden. Some students wanted to move back to their home city. They already had plans for their future jobs, and needed the location of the job to be near to their homes. Others wanted to pursue a higher academic degree after a short-term job. This was reflected by one student (nHS2-34) who said, “I had the pressure of repaying the money if I signed the contract and then didn’t stay. I live in Shi-Pai and my family hoped my work location would be close to my home.” Others had decided to take a short-term job and pursue a higher academic degree. Signing a contract would limit their flexibility.

I plan to go to graduate school at National University School of Nursing -B (SN-B), so it’s more convenient and closer for me to work at the SN-B hospital. If I signed the contract, it would affect my plans for the future. (Student nHS1-1)

##### Evaluation of the job environment and location (HS:58%, nHS:43%)

The job environment and location were significant factors in the decision-making process for 58% of hospital-sponsored students, and only 43% of the non-sponsored students. Familiarity with the teaching hospital providing the scholarship was an important consideration for the hospital-sponsored students. Students understood the clinical setting, the work environment and the corporate culture of the hospital. One student (HS1-9) had also considered her family’s opinion of the hospital when making her decision saying, “My family was very supportive because they had a good impression of the corporation that runs the hospital and the hospital’s in the community. Another student made the decision to participate in the program in order to work in a hospital located in an area of Taiwan that was familiar, “I think I was afraid of change and a new environment. So, if I could stay in a familiar place I would feel more secure.” (HS2-27).

In contrast, non-sponsored often made the decision to decline the scholarship because aspects of the environment were seen as negative, including the hospital, the climate. Some non-sponsored students could not identify with the corporate culture or work environment. One student could not tolerate the city or the weather, saying, “I don’t like the city. The weather is really humid and cold and with strong winds. I have not gotten used to it since the first day I enrolled in the school.” (nHS2-31) One non-sponsored student’s decision was influenced by the parent’s negative views of the hospital environment, who said, “Frankly speaking, whether it was sponsored by the hospital or not, my parents believe National medical center-B (H-B) is much better than H-A. Although they are both academic medical centers.” (nHS1-1) Another student (nHS2-22) did not like the culture of the hospital:

I live in Taipei and my family wanted me to find a job in an academic medical center in Taipei. For myself, I do not want to stay at this hospital (H-A) because I’ve witnessed the seniors being disrespectful to new recruits, which I think is mean. But they were ok with the interns. Also, people refer to this hospital the overworking hospital.”

##### Evaluation of employment outcomes

Hospital-sponsored students (47%) decided to accepted the scholarship program because they were confident they could fulfill the contract. For some, the positive impression of the training provided by the sponsoring hospital gave them confidence.

For me, I wanted to work in a big hospital as soon as I graduated because you can learn more and gain more experience. It was essential to have working experiences in academic medical centers. Since I am familiar with H-A, it would be best to stay here. (HS1-4)

Some hospital-sponsored students had confidence they would complete the contract because of parental expectations that they fulfill their obligation. Student HS1-2 said, “Once I decided to sign the contract, my parents felt relieved and they told me that I must persist with the work through the full-term.” A second student explained the decision as follows:

Well, I signed the five-year contract but I plan to work here for six years. Although I am still worried that I may not survive this challenge I must hang in here! Since I signed the contract, I must fulfill my responsibility and keep going forward! (HS2-37)

Evaluation of employment options was a factor in deciding on the scholarship for 34% of non-sponsored students. For these students, family members believed employment in the hospital that sponsored the scholarship would not provide the type of experience necessary for a secure job in the future. Families of non-sponsored students believed work experience in academic medical centers would ultimately lead to a government job, which was more secure. One student, who planned to take the exam for graduate school said, “.. .my parents thought it would be better to go to H-B because it is a medical center and I can work as a government official one day. I thought my future would be more secure.” (Student nHS1-1) Similar parental reactions were shared by another student (nHS2-33), who said, “My father does not support me in this program, he thought it would be better to go to H-B, because it is the best hospital in Taiwan.” Work at a medical center was also important to another student’s parents:

My parents hoped that I could work in an academic medical center. On one hand, it was near my home to go to National medical center-C (H-C). On the other hand, I might have the chance to apply for a government position, which would be more solid and I would feel more settled. (Student nHS2-34)

#### Analyzing the decision

Whether they made the choice to accept or decline the scholarship, both groups of students had doubts about their decision. They experienced anxiety and uncertainty, and employed coping mechanisms to help them validate their decision. They also shared their perspectives about the hospital-sponsored scholarship.

##### Anxiety and uncertainty

Nearly all the hospital-sponsored students (90%) experienced anxiety and uncertainty about accepting the scholarship. This was due to doubts and worries about their ability to complete the contract, which stemmed from concern about whether their personality would be compatible with peers, or whether they would be viewed differently because of the scholarship. One student (HS1-2) said, “Of course I am worried! I do not voluntarily tell others that I am a sponsored student because I am afraid they might see me differently.” Another said,

I’m scared to death!. .. (I am) worried that if I cannot get along well with the colleagues in my unit, I will not survive. After signing the contract and getting the money, it turned out that you cannot even stay and serve in the unit … gosh! Tell me what to do then. (HS2-40)

They feared their identity as a sponsored student would prevent them from voicing wishes about job placement, or might have a negative impact on income or training.

I am worried because we are not the same as those who were recruited in the normal way. We might get a smaller bonus or reward, or the training might be different. I am worried I will not be treated equally, just because I got the money first. I worry that people will assign us (to a unit) or ask us do whatever they want just because we are sponsored students. I worry I will not have a choice of hospital unit, and this is the thing I fear the most. (HS1-4)

Only 41% of non-sponsored students described feelings of anxiety and uncertainty about their decision. Similar to hospital-sponsored students, some non-sponsored students were concerned about being accepted by their peers. One student said, “I am an introvert and I am really worried that I cannot get used to the new working environment or how to interact with the seniors at the H-C. This bothers me a lot.” (nHS2-32) In contrast to hospital-sponsored students, non-sponsored students were worried about job placement or the lack of information about their new work environment. One student, who was waiting to hear about the results of the qualifying exam at H-B, said,

I felt really uncertain about things while waiting for the confirmation and actually school is quite different from the clinical workplace. Every hospital has their own rules and cultures and I am quite worried that I cannot get used to that. (nHS2-33)

Another student described worrying about entering a new job with no information about the hospital environment:

I did not know much about other hospitals. Every hospital’s circumstances and conditions are not the same and it takes a lot of time asking others and figuring things out; I don’t know whom I should ask. Not knowing where to work and not knowing anything really troubles me. (nHS2-28)

##### Coping with the decision

Coping mechanisms were employed to reduce anxiety and uncertainty following the decision by nearly twice as many hospital-sponsored students (54%) than non-sponsored student (28%). Both adopted a positive attitude about their future, but the focus of this motivation differed for the two groups. Hospital-sponsored students began making plans to prepare for the possibility of not securing a permanent position in their unit upon completion of their contract. Students who were worried about job stability decided to save the money received upfront in case they were not retained by the hospital or they needed to transfer to a different hospital unit.

I’m like my classmates who plan to get the money from the sponsorship first and never ever spend it. If we cannot survive throughout the contract or do not want to work anymore, we can pay back the exact amount of money. Hahaha, it is like getting the money first and pondering the situation later. (HS1-4)

Hahaha, I think I will do like what my senior did and save the money for a while. If I am sure that I can stay here without any problem, then I will spend it. But if I do not fit the job, I do not think I will quit. I will probably think about changing to a different unit. I think this is a more practical approach. (HS2-40)

Non-sponsored students were free from the pressure of a contract. If they encountered problems fitting into the work environment, they reminded themselves that they could quit anytime. They also believed they could return to their original hospital (H-A), which was a familiar place. One non-sponsored student said, “If I cannot get used to the work environment at H-C, I might return to H-A, at I am familiar with it. But first I want to try other hospitals at the beginning.” (nHS2-23) A student who needed to complete military service said, “If other hospitals are not suitable or I cannot fit in well, I think there is a great chance that I will want to be back to the original hospital (H-A).” (nHS2-35).

### Perspectives of the hospital-sponsored scholarship

Participants were asked to share their perspectives about the hospital-sponsored program. Sponsored students provided suggestions for how the program might be improved for future students. They felt the presentation of the program should be restructured in order to provide more specific details about the benefits of the program. Sponsored students (51%) believed there were some inconsistencies in the policy and they questioned whether the selection process was fair to all students. One sponsored student said, “No one explained the details well. I had difficulty understanding who needed to fill-out parts of the application form. I think the hospital should explain the specific procedures, for example, what kind of information I should fill-in, who should fill these.. .” (HS1-4) Another student thought the school and hospital should have been more unified in their goals and presented the program to the students earlier, which would increase the benefits of the internship.

In my class year, students were asked if they had opinions about their choice of internship. But some students did not know they had a choice about what they wanted and they complained a lot. I think it is not good if some were asked and some were not. (HS2-37)

The program was viewed as an effective way to attract good and talented people to enroll in the nursing program by 47% of students who were hospital-sponsored compared with only 24% of non-sponsored students. One student said, If the schools’ levels are similar, I will prioritize those school that provide scholarships. It will totally change my rank of schools because I cannot only get the money but also the job. (HS1-2) Another thought the sponsorship ensured a smooth transition from school to the healthcare setting, saying

The program will help us know more about working in a clinical setting; possibilities of fainting when working may happen less. We can choose the units we are interested in when deciding our internship and when it comes time to work there. I think our expectations will be more realistic. (HS2-37)

The non-sponsored students felt the program would not increase retention of new nursing professionals unless there was a significant change in the hospital work environment.

But I still feel it (the program) is not the ultimate solution to the problem. The hospitals have problems keeping nurses from leaving, so they use this contract to keep them for three or five years. But they never considered improving the overall working environment, and this program will never be the best way to solve the problem. But for those who need employment and money, it is a good way to bind them in the hospital. (nHS1-1)

## Discussion

This qualitative descriptive study explored the experiences of new nursing school graduates who were presented with the option of participating in a hospital-sponsored scholarship program. All students underwent a decision-making process when evaluating whether to accept or reject the scholarship: 1) considering options, 2) making the decision, and 3) analyzing their decision. The primary factors that influenced the outcome were financial status, the hospital environment, and consideration of future long-term career goals.

As students considered their options prior to signing the hospital contract, both sponsored and non-sponsored students reacted with surprise and disbelief because the program seemed too good to be true and they sought out the opinions of senior nursing graduates with clinical experience. However, for the students in the graduating class of 2013, there were no previous nursing graduates who had been offered the opportunity of participating in the hospital-sponsored scholarship program. In contrast, participants in the graduating class of 2014 were able to obtain opinions of HS1 and nHS1 nurses from the class that had graduated in 2013. However, students who ultimately accepted the scholarship were those that were able to not only obtain details of the program, but also received positive feedback from friends and family members, which they described as a critical factor in their decision-making. In contrast, non-sponsored students did not mention making an effort to obtain additional information about the program and did not receive seek encouragement from others to make their decision. The strong influence of parents when students were considering their options is similar to a systematic review of the literature showing the importance of family in nursing career decisions [19]. In addition, quantitative and qualitative studies report parental expectations, approval, and support from family were significant factors regarding nursing career decisions for students in Taiwan [16, 28, 29].

The decision to accept or reject the scholarship included evaluating the financial benefits of a long-term commitment to one hospital and the obligation to pay back the signing bonus should the nurse chose to leave before the end of the contract. Most students who accepted the scholarship did so to relieve the financial burden they had incurred as a student. In contrast, for the non-sponsored students, financial incentive was not a factor in committing to the program because they had no financial burden. Thus, the payback requirement was the primary motivator when non-sponsored students evaluated the financial impact. These findings are similar to previous studies that examined the impact of financial incentives on increasing retention of nurses [17, 19, 30], indicating salary and financial need significantly influenced choice.

The hospital environment was a more important factor for the sponsored than the non-sponsored students, and students’ families supported this decision. Sponsored students had enjoyed their clinical rotations in the hospital and were comforted by familiarity with the environment and culture of the hospital; the close proximity of the hospital to the students’ homes was also a positive factor. These findings are similar to a study by Liang, Lina and Wud [31] who reported familiarity with the hospital environment, work culture, and knowledge of job expectations were important when newly graduated Taiwanese nurses were faced with long-term career choices, which reduced the stress of a new job. For the non-sponsored students long-term career goal influenced their decision to decline the scholarship. Many non-sponsored students had already made plans to enroll in advanced-degree programs at other universities, and did not want any future career decisions to be constrained by the terms of the contract. Their families supported this decision because they believed other hospitals had a better reputation and offered more and better career opportunities. These findings are similar to a qualitative interview study by Dos Santos [32] with 42 fourth-year Taiwanese nursing students, who reported students’ choices for career pathways were influenced by work environment, family recommendations and filial piety.

Once the decision had been finalized, both sponsored and non-sponsored students experienced anxiety and worry about being new graduates and their new working situations. Both hospital-sponsored and non-sponsored students experienced anxiety about working with a new group of peers and worried about fitting in with these new nurses. Sponsored students knew what to expect regarding the job culture, but they would be working with new people in a yet-to-be-determined hospital unit. The non-sponsored students who had not established a clear path for their future had no idea what their work environment would be. Sponsored students worried they might be treated differently if others knew they were recipients of the scholarship. Australian nursing students who were paid to work prior to graduation experienced similar anxieties [13].

## Study limitations

In spite of its strengths, this study had some limitations. Not all students who met the inclusion criteria and agreed to participate were interviewed because they returned home to prepare for their licensing exam. We do not know if including interview data for these students would have altered our findings. A second limitation is the class of 2014 had an opportunity to obtain information from the hospital-sponsored students from the class of 2013. This was not an option for the students in the class of 2013. Therefore, the second class may have had a better understanding of the sponsorship and might have experienced less anxiety and worry about making the decision. Third, there were only five male students (6%); three were required to complete their military service immediately after graduation and did not need to find a job immediately. The small sample size of male nurses prevents assessing whether gender had an impact on the responses of the students. Finally, the data were collected in 2013 and 2014, and is over 5 years old. However, the hospital-sponsored scholarship program continues to be offered to current nursing students in Taiwan. The conditions and qualifications of the recruitment program and the amounts offered have not changed.

## Conclusion

Increasing retention is one avenue of addressing the shortage of nurses in the healthcare industry. The hospital-sponsored scholarship program offered in Taiwan did not appeal to all students. The hospital-sponsored students were more likely to have financial needs while the non-sponsored students were influenced by a desire to pursue a more academic career that would provide long-term job security. When asked if they believed the program would improve retention, students suggested it would reduce uncertainty about what to expect, but it was more important to improve the work environment. Quantitative studies will be required to determine if these types of collaborations between hospitals and academia can increase retention for new nurse graduates.

## Data Availability

The datasets used and/or analysed during the current study are available from the corresponding author on reasonable request.

## Abbreviations

IRB: Institutional Review Board
SN-A: Private University School of Nursing
H-A: Collaboration with a Private Medical Center
GPA: Grade Point Average
PhD: Doctor of Philosophy
HS: Hospital-sponsored scholarship
nHS: Non-hospital sponsored
NTD: New Taiwan dollar
RN: Registered Nurse
H-A: Academic Medical Center in Taipei
H-B: National Medical Center-B
H-C: National Medical Center-C
